# P/Q and N-type Voltage-gated Calcium Channel Binding Antibodies Associated with Paraneoplastic Chorea and Mixed Invasive Ductal and Lobular Carcinoma of the Breasts in an Elderly Patient

**DOI:** 10.7759/cureus.3097

**Published:** 2018-08-04

**Authors:** Kevin Chang, Anita Lwanga, Tanjeev Kaur, Cathy Helgason

**Affiliations:** 1 Division of Academic Internal Medicine and Geriatrics, University of Illinois at Chicago, Chicago, USA; 2 Department of Academic Internal Medicine and Geriatrics, University of Illinois at Chicago, Chicago, USA; 3 Department of Medicine/Division of Academic Internal Medicine and Geriatrics, University of Ilinois at Chicago, Chicago, USA; 4 Department of Neurology, University of Ilinois at Chicago, Chicago, USA

**Keywords:** chorea, paraneoplastic syndrome, paraneoplastic chorea, n type voltage gated calcium channel binding antibodies, p/q type voltage gated calcium channel binding antibodies, paraneoplastic antibodies, breast cancer

## Abstract

Paraneoplastic neurologic syndromes are a group of immune-mediated, cancer-associated disorders affecting the nervous system. While these syndromes are not understood fully, they are reportedly caused by an immune response against common antigens expressed by the cancer and nervous system. We describe the course of a patient who suffered paraneoplastic chorea before being diagnosed with breast cancer. A 70-year-old female presented with complaints of “shaking” movements of her head. History, physical exam findings, and preliminary workup ruled out the hereditary, metabolic, and infectious causes of chorea while brain computed tomography (CT) ruled out chorea due to a basal ganglia lesion. A paraneoplastic antibody panel identified N-type and P/Q-type voltage-gated (V-G) calcium channel binding antibodies. Subsequent age-appropriate cancer screening, which included a colonoscopy and screening mammograms, identified breast cancer. The patient had bilateral total mastectomies. Histopathology confirmed mixed invasive ductal and lobular carcinoma that was estrogen receptor positive, progesterone receptor positive, and human epidermal growth factor receptor 2 negative. In addition to mastectomies, the patient received adjuvant anastrozole. The appearance of choreiform movements before the diagnosis of breast cancer and the presence of paraneoplastic antibodies indicated that the chorea was most likely paraneoplastic in nature. Our patient continues to have choreiform movements despite undergoing bilateral mastectomies and receiving anastrozole, prednisone, and rituximab. We suspect the mastectomies and immune modulating therapies have not had an effect on her chorea because her P/Q and N-type V-G calcium channel binding antibodies may be intracellular. This case of paraneoplastic chorea associated with breast cancer is unusual. To the best of our knowledge, only one other case of paraneoplastic chorea associated with breast cancer has been reported in the English literature.

## Introduction

Paraneoplastic neurologic syndromes are a group of immune-mediated, cancer-associated disorders affecting the nervous system [1]. While the mechanisms underlying these syndromes are not understood fully, they may be caused by an immune response against common antigens expressed by the cancer and nervous system [1]. Well-known paraneoplastic syndromes include P/Q-type voltage-gated (V-G) calcium channel antibodies in Lambert-Eaton myasthenic syndrome [2] and N-methyl-D-aspartate (NMDA) receptor antibodies in anti-NMDA receptor encephalitis [3].

The incidence of paraneoplastic syndromes varies with the type of tumor. A common paraneoplastic syndrome is myasthenia gravis, occurring in approximately 15% of individuals with thymoma [4]. For most solid tumors, the paraneoplastic disorders are less common, with an incidence of <1% [5].

We describe the course of an elderly patient with paraneoplastic chorea associated with breast cancer.

## Case presentation

In December 2012, a 70-year-old woman presented with the chief complaint of “shaking” head movements. The movements began one month previously, shortly after she began taking hydroxychloroquine for seropositive rheumatoid arthritis (RA).

In addition to RA, the patient had a past medical history of atrial fibrillation, cardioembolic cerebrovascular accident, two transient ischemic attacks, Sjögren’s syndrome, pseudogout, calcium pyrophosphate disease, osteoarthritis, pacemaker placement, left-sided multinodular goiter, osteoporosis, chronic hypertension, heart failure with preserved ejection function, recurrent bilateral lower extremity deep vein thromboses, iron deficiency anemia, anxiety disorder, major depressive disorder, and dyslipidemia. She did not smoke tobacco, drink alcohol, or use illicit drugs. She danced once or twice a week to maintain physical fitness. Her family history was not contributory. She did not have allergies. Her medications included atorvastatin, carvedilol, cyclosporine, docusate, ergocalciferol, ferrous sulfate, folic acid, furosemide, losartan, melatonin, methotrexate, omeprazole, polyethylene glycol, prednisone, hydroxychloroquine, and warfarin.

Blood pressure was 135/72 mmHg, temperature was 97.7 Fahrenheit (38.6 Celsius), and body mass index was 41.05 kg/m^2^. She was alert and oriented to person place and time, and not in acute distress. Cranial nerves 2–12, and sensation to pinprick, vibration, and joint position were intact. Reflexes were 2+ at all the tendons, and strength was 5/5 in all the extremities. Gait was ataxic and she had choreiform movements affecting her head, upper extremities, and lower extremities. There was some dysmetria with finger-to-nose testing. The heart had normal rate and rhythm with a holosystolic murmur in the aortic region. The pulmonary, abdominal, and integumentary exams were unremarkable.

Arrangements were made to contact the patient's rheumatologist to determine if the head movements were related to hydroxychloroquine and obtain medical records from outside facilities. Results from a computed tomography (CT) scan of the head performed elsewhere four months previously were unremarkable except for some abnormalities around the pituitary region.

By February 2013, the truncal ataxia and abnormal head movements were still present. Hydroxychloroquine was halted, and rituximab was started, in case the former medication was the cause of her abnormal movements; however, the movements continued. Complete blood count (CBC), complete metabolic panel (CMP), thyroid function tests (TFTs), parathyroid hormone, lipid panel, vitamin B12 level, human immunodeficiency virus (HIV) screen, rapid plasma reagin, iron panel with ferritin, and creatine kinase (CK), were all within the normal limits. Antinuclear antibody, anti-Ro antibodies, rheumatoid factor, and anti-cyclic citrullinated peptide were positive; anti-La antibody was negative. A paraneoplastic antibody panel demonstrated P/Q and N-type V-G calcium channel antibodies. Table 1 shows the full results of the paraneoplastic antibody panel. By November 2013, the cerebellar ataxia had resolved; however, the patient reported occasional sensations of feeling like she was “on a ship” and had some difficulties maintaining her balance.

**Table 1:** Results of the patient’s paraneoplastic antibody panel Legend: Ab: antibody; AChR: acetylcholine receptor; CRMP-5: collapsing response-mediator protein; V-G: voltage gated

Test	Result	Reference Range
Antineuronal Nuclear Ab, Type 1	Negative titer	< 1:240
Antineuronal Nuclear Ab, Type 2	Negative titer	< 1:240
Antineuronal Nuclear Ab Type 3	Negative titer	< 1:240
Anti-Glial Nuclear Ab Type 1	Negative titer	< 1:240
Purkinje Cell Cytoplasmic Ab Type 1	Negative titer	< 1:240
Purkinje Cell Cytoplasmic Ab Type 2	Negative titer	< 1:240
Purkinje Cell Cytoplasmic Ab Type Tr	Negative titer	< 1:240
Amphiphysin Ab	Negative titer	< 1:240
CRMP-5-IgG	Negative titer	< 1:240
Striational Striated Muscle Ab	Negative titer	< 1:60
P/Q-Type V-G Calcium Channel Ab	0.04	<= 0.02
N-Type V-G Calcium Channel Ab	0.06	<= 0.03
AChR Muscle Binding Ab	Negative titer	<= 0.02
AChR Ganglionic Neuronal Ab	Negative titer	<= 0.02
Neuronal V-G Potassium Channel Ab	Negative titer	<= 0.02

**Figure 1:**
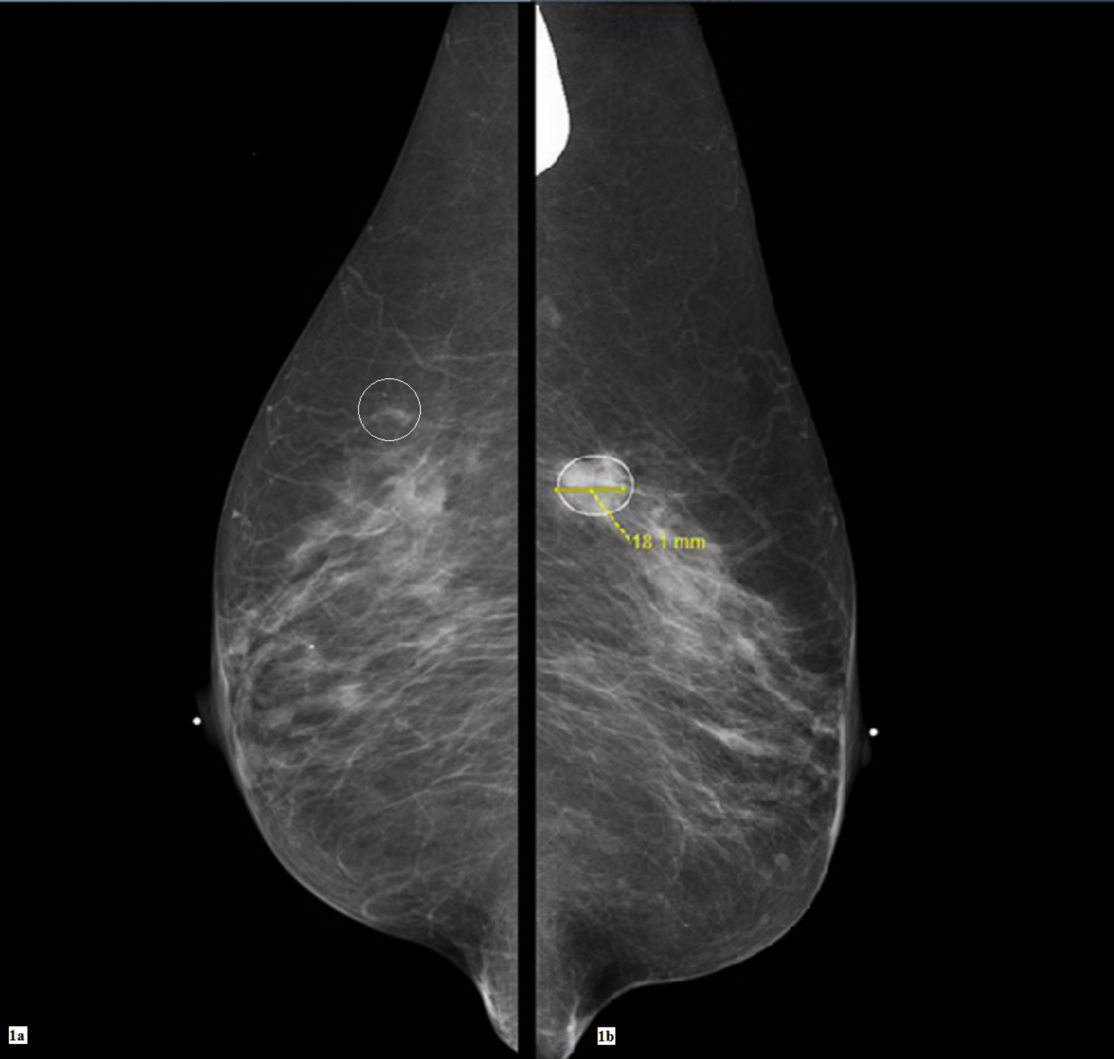
Screening bilateral digital mammograms In Figure 1a at the upper quadrant at the 11:00 axis of the right breast, there is a subtle area of focal asymmetry. In Figure 1b at the upper outer quadrant of the left breast around the middle third depth, at the 2:00 axis, an irregular mass can be seen.

**Figure 2:**
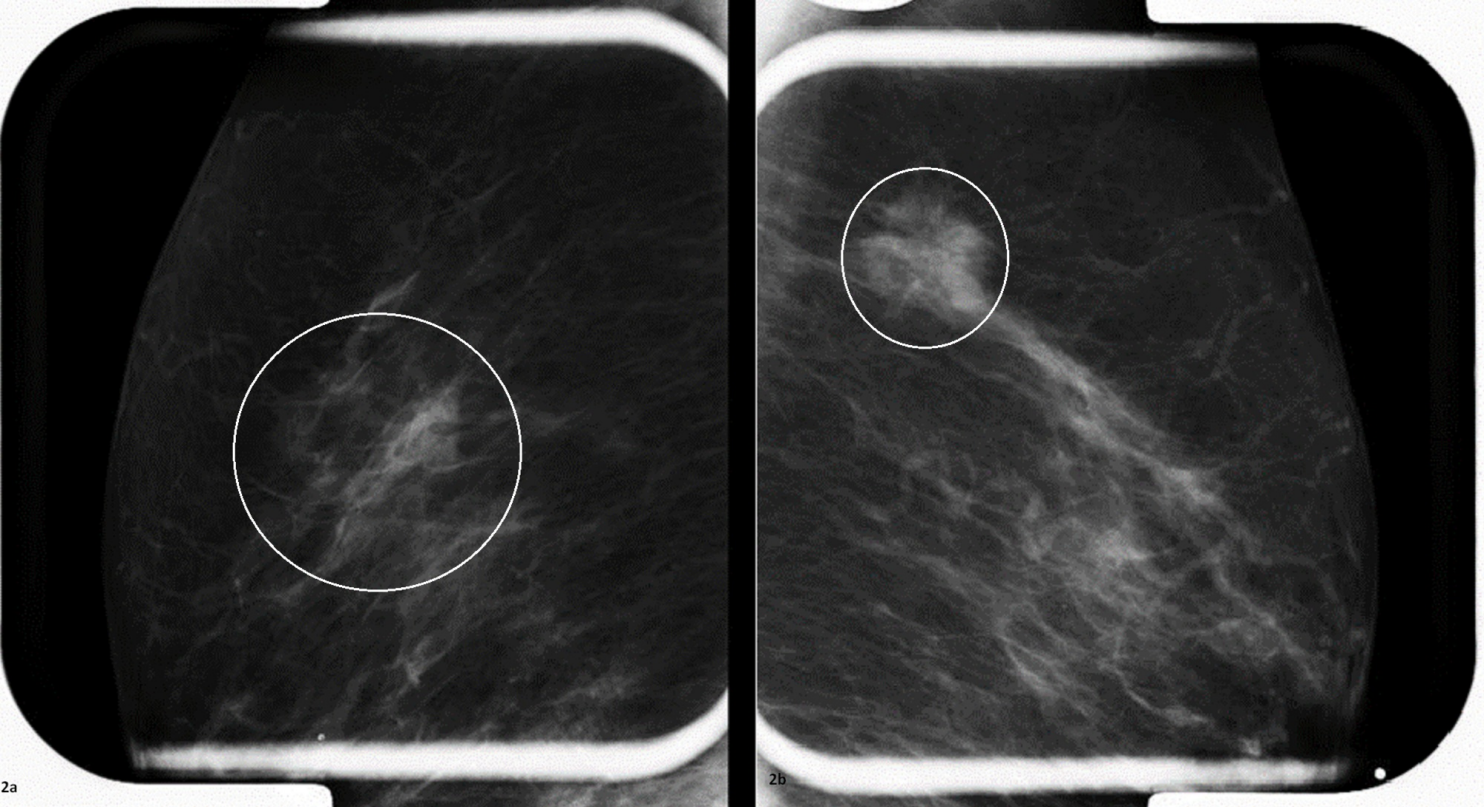
Spot compression and magnification views The area of focal asymmetry at the right breast is still visible with spot compression (Figure 2a). The mass at the left breast (Figure 2b) appears to be about 2 cm in diameter in the spot compression view.

In January 2014, the cerebellar ataxia returned prompting a workup for malignancy. Based on the US Preventative Task Force's recommendations about age-appropriate cancer screening for a 70-year-old-female a colonoscopy and screening mammograms were ordered. The colonoscopy was only remarkable for diverticulosis. Bilateral screening mammograms (Figures 1-2) and ultrasounds done in April 2014 identified a 2.1 × 2.0 × 1.7 centimeter (cm) mass and a 0.7 × 0.5 cm hypoechoic area at the upper outer quadrants of the left and right breasts, respectively. Two months later, bilateral core needle biopsies confirmed bilateral breast cancer. 

In September 2014, she presented again with complaints of intermittent abnormal movements of the head, with choreiform and athetoid characteristics; the movements were absent at rest and present with movement. In the same month, she underwent total bilateral mastectomies. The left breast specimen was 2.2 × 1.6 × 1.4 cm with a negative sentinel lymph node, and that from the right breast was 1.1 × 1.0 × 0.6 cm, with three negative lymph nodes. Histopathology revealed mixed invasive ductal and lobular carcinoma. Immunohistochemistry was weakly estrogen receptor (ER) positive, weakly progesterone receptor (PR) positive, and human epidermal growth factor 2 (HER2) negative. Antigen Ki-67 results predicted a low risk of recurrence. Tumor protein P53 was positive in the left breast but negative in the right breast.

In April 2015, she continued to have intermittent episodes of abnormal movements that also were unresponsive to trails of gabapentin and clonazepam.

## Discussion

Chorea is a hyperkinetic movement disorder characterized by brief, nonrepetitive, irregular, involuntary movements of the limbs, trunk, neck, or face [6]. It can be hereditary (such as in individuals with Huntington’s disease), metabolic (such as in those with thyroid disorders), autoimmune (such as in lupus patients), medication-induced (which can be caused by levodopa), infectious (such as in individuals with HIV/acquired immunodeficiency syndrome (AIDS)), because of a structural lesion (such as in individuals with a stroke affecting the basal ganglia), or paraneoplastic (such as in some cases of lung cancer) [6].

For patients presenting with chorea, their history and physical exam may be used to generate a differential diagnosis. Additional tests, such as CBC, CMP, TFTs, parathyroid hormone level, autoimmune panel, HIV test, syphilis screen, iron panel with ferritin, creatine kinase (CK), paraneoplastic antibody panel, genetic testing, electroencephalogram, CT or magnetic resonance imaging (MRI) of the brain, and/or lumbar puncture may be indicated [6]. The results of these tests may reveal the cause of the chorea; however, in many cases, despite extensive testing, the diagnosis remains elusive [6].

We reported on an elderly woman with head and extremity choreiform movements who did not have a family history of chorea; therefore, it was unlikely that her chorea was hereditary. She was unable to undergo a brain MRI because she had a pacemaker. Nevertheless, blood work and brain CT scan ruled out chorea because of hypo/hyperglycemia, hypo/hypernatremia, hypo/hypercalcemia, neuroacanthocytosis syndromes, Wilson’s disease, hypo/hyperthyroidism, hypo/hyperparathyroidism, McLeod syndrome, vitamin B12 deficiency, ataxia with oculomotor apraxia 1 and 2, or basal ganglia lesion [6]. Based on the initial evaluation, Sjögren’s syndrome, an adverse reaction to medications, and paraneoplastic chorea were considered possible causes of her symptoms.

Sjögren’s syndrome usually is characterized by lacrimal and salivary gland dysfunction, but it can manifest also with extraglandular features, including chorea [6-7]. In Sjögren’s syndrome, if the anti-Ro and anti-La antibodies cause damage to the basal ganglia, the patient can have chorea, which would be classified as autoimmune in nature [6]. Our patient was diagnosed with Sjögren’s syndrome, with anti-Ro antibodies, before the chorea developed. If her chorea was because of Sjögren’s syndrome, the chorea should have developed before or around the time she was diagnosed with the condition, not afterwards. Furthermore, the presence of P/Q and N-type V-G antibodies could not be explained by an autoimmune condition since these antibodies are seen in paraneoplastic conditions [6]. The presence of paraneoplastic antibodies should prompt the clinician to look for an occult malignancy as was the case in our patient. Vengas et al. described the course of a patient who presented with generalized chorea as the initial manifestation of Sjögren’s syndrome. The chorea involved axial musculature and the face, with subsequent development of depressive symptoms and mild cognitive impairment [8]. However, that patient differs from our patient in that the former had no comorbid diagnosis of breast cancer and the case report does not mention paraneoplastic antibodies.

Since our patient suffered chorea after she started taking hydroxychloroquine, medication-induced chorea was considered. Owing to the lack of resolution of her symptoms after discontinuing the medication, it became obvious that hydroxychloroquine was less likely the cause of her chorea. Later, it was thought that the movements may have been exacerbated by gabapentin; however, in spite of discontinuing this medication, her symptoms persisted.

Paraneoplastic chorea is rare and has been associated with small cell lung cancer and, less commonly, with thymomas, testicular cancer, and breast cancer [9-11]. Many patients with paraneoplastic chorea present with concurrent vision loss, limbic encephalitis, or ataxia [12], similar to our patient. In our case, the paraneoplastic antibody panel results indicated that she may have had a paraneoplastic process because of an occult malignancy [6]; however, the underlying cause was not immediately obvious. Owing to the presence of P/Q and N-type V-G antibodies, a workup was done for malignancy, and stage T2/N0/M0 carcinoma with ductal and lobular features was diagnosed; immunohistochemistry was ER positive, PR positive, and HER2 negative. Since this is the only malignancy that she had, it is most likely the cause of her paraneoplastic chorea.

To our knowledge, only one case of paraneoplastic chorea associated with breast cancer has been reported in the English literature [9]. The report described the course of a 60-year-old female who initially presented with acute schizoaffective psychosis, which was managed with neuroleptics and electroconvulsive therapy. Two weeks later, choreiform movements gradually appeared on her left side. Two months later, ductal carcinoma in situ of the left breast was diagnosed. She underwent mastectomy, followed by anastrozole. Though her cancer was treated effectively, later she was admitted to a movement disorder center for the evaluation of persistent choreiform movements. Genetic testing ruled out Huntington’s chorea and Wilson’s disease; a paraneoplastic antibody panel identified anti-Hu and anti-Ri antibodies, which confirmed that her chorea was paraneoplastic in nature. The patient received amantadine sulfate and intravenous methylprednisolone, neither of which had any effect on the chorea. The authors postulated that her treatment failed because the anti-Hu and anti-Ri antibodies were intracellular [9].

In classic paraneoplastic encephalitidies, the neuronal damage usually is rapid and irreversible [13]. Unfortunately, despite maximal treatment, these patients rarely have a complete resolution of their neurologic symptoms [14]. In these cases, the best chance for meaningful recovery is treating the primary tumor and administering immunotherapy with glucocorticoids, intravenous immunoglobulin (IVIG), plasma exchange, cyclophosphamide, and rituximab [13-15]. If the antibodies are extracellular, immunotherapy may remove or reduce the number of antibodies; if the antibodies are intracellular, immunotherapy is less likely to be effective [9]. Like the patient described above, our patient has ongoing choreiform movements despite undergoing bilateral mastectomies and receiving anastrozole. She has been taking prednisone and rituximab, primarily to treat her RA; however, these immune modulating therapies have not had an effect on her chorea. She is in remission and has not been diagnosed with another malignancy. Like the patient described by Martinková et al. [9], our patient may have persistent symptoms because her P/Q and N-type V-G calcium channel binding antibodies are intracellular.

## Conclusions

This patient’s presentation of paraneoplastic chorea associated with bilateral ER positive, PR positive, HER2 negative breast carcinoma with ductal and lobular features is unusual. To the best of our knowledge, only one other case of paraneoplastic chorea associated with a diagnosis of left-sided ductal carcinoma in situ has been reported in the English literature.
